# Supplementary material for: Causal Relationship between Aging and Anorexia Nervosa: A White-Matter-Microstructure-Mediated Mendelian Randomization Analysis
Source: Biomedicines. 2024 Aug 16;12(8):1874. doi: 10.3390/biomedicines12081874 (PMC11351342; doi:10.3390/biomedicines12081874)
Supplement: Supplementary file 1 [file biomedicines-12-01874-s001.zip › Supplementary Figure/Figure S4/Singe SNP plot of MR analysis for TL to ubm-b-1835.pdf]

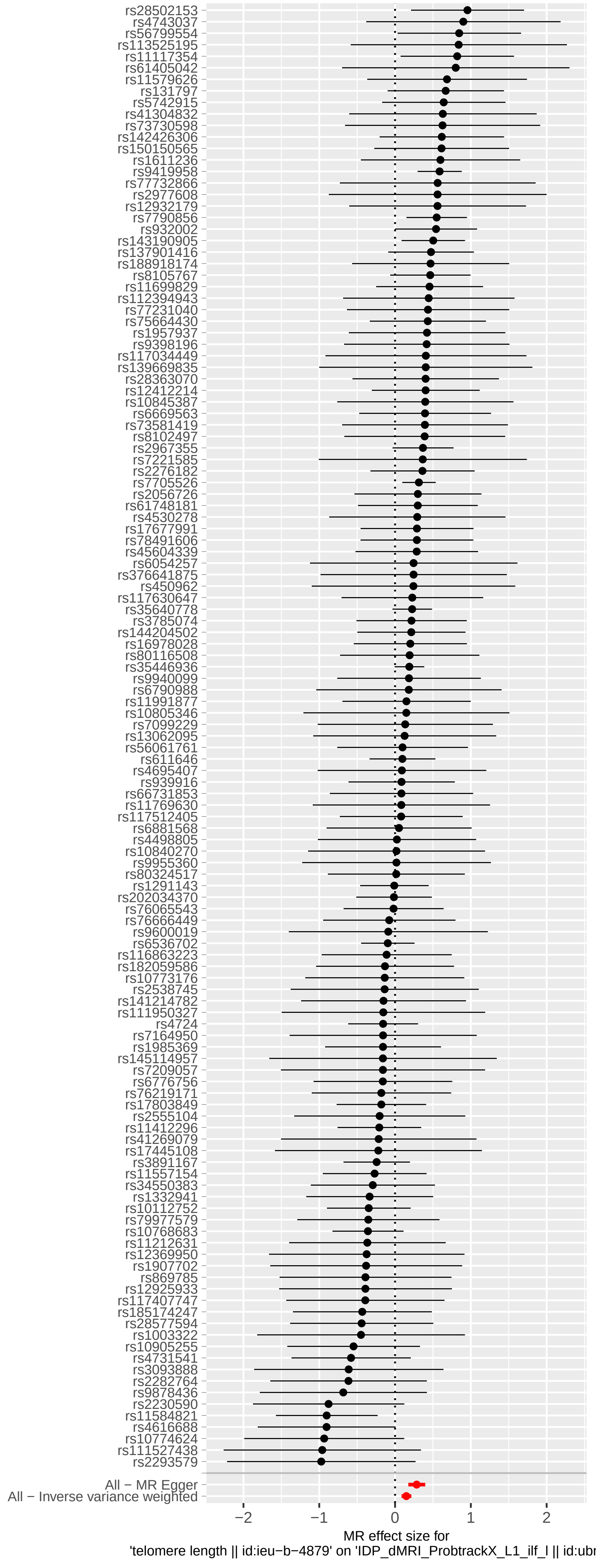

rs28502153
rs4743037
rs56799554
rs113525195
rs11117354
rs61405042
rs11579626
rs131797
rs5742915
rs41304832
rs73730598
rs142426306
rs150150565
rs1611236
rs9419958
rs77732866
rs2977608
rs12932179
rs7790856
rs932002
rs143190905
rs137901416
rs188918174
rs8105767
rs11699829
rs112394943
rs77231040
rs75664430
rs1957937
rs9398196
rs117034449
rs139669835
rs28363070
rs12412214
rs10845387
rs6669563
rs73581419
rs8102497
rs2967355
rs7221585
rs2276182
rs7705526
rs2056726
rs61748181
rs4530278
rs17677991
rs78491606
rs45604339
rs6054257
rs376641875
rs450962
rs117630647
rs35640778
rs3785074
rs144204502
rs16978028
rs80116508
rs35446936
rs9940099
rs6790988
rs11991877
rs10805346
rs7099229
rs13062095
rs56061761
rs611646
rs4695407
rs939916
rs66731853
rs11769630
rs117512405
rs6881568
rs4498805
rs10840270
rs9955360
rs80324517
rs1291143
rs202034370
rs76065543
rs76666449
rs9600019
rs6536702
rs116863223
rs182059586
rs10773176
rs2538745
rs141214782
rs111950327
rs4724
rs7164950
rs1985369
rs145114957
rs7209057
rs6776756
rs76219171
rs17803849
rs2555104
rs11412296
rs41269079
rs17445108
rs3891167
rs11557154
rs34550383
rs1332941
rs10112752
rs79977579
rs10768683
rs11212631
rs12369950
rs1907702
rs869785
rs12925933
rs117407747
rs185174247
rs28577594
rs1003322
rs10905255
rs4731541
rs3093888
rs2282764
rs9878436
rs2230590
rs11584821
rs4616688
rs10774624
rs111527438
rs2293579
All − MR Egger
All − Inverse variance weighted
−2
−1
0
1
2
MR effect size for
'telomere length || id:ieu−b−4879' on 'IDP_dMRI_ProbtrackX_L1_ilf_l || id:ubr
